# Characterization of a New Reconstructed Full Thickness Skin Model, T-Skin™, and its Application for Investigations of Anti-Aging Compounds

**DOI:** 10.3390/ijms20092240

**Published:** 2019-05-07

**Authors:** Michel Bataillon, Damien Lelièvre, Adeline Chapuis, Fabienne Thillou, Jean Baptiste Autourde, Steven Durand, Nathalie Boyera, Anne-Sophie Rigaudeau, Isabelle Besné, Christian Pellevoisin

**Affiliations:** EPISKIN SA, 4, rue Alexander Fleming, 69366 Lyon, France; mbataillon@episkin.com (M.B.); dlelievre@episkin.com (D.L.); achapuis@episkin.com (A.C.); fchapuis@episkin.com (F.T.); jbautourde@episkin.com (J.B.A.); sdurand@episkin.com (S.D.); nboyera@episkin.com (N.B.); asrigaudeau@episkin.com (A.-S.R.); ibesne@episkin.com (I.B.)

**Keywords:** T-Skin, full-thickness skin, reconstructed skin, anti-aging, characterization, vitamin C, retinol

## Abstract

Background: We have characterized a new reconstructed full-thickness skin model, T-Skin™, compared to normal human skin (NHS) and evaluated its use in testing anti-aging compounds. Methods: The structure and layer-specific markers were compared with NHS using histological and immunohistological staining. In anti-aging experiments, T-Skin^TM^ was exposed to retinol (10 µM) or vitamin C (200 µM) for 5 days, followed by immunohistological staining evaluation. Results: T-Skin™ exhibits a well stratified, differentiated and self-renewing epidermis with a dermal compartment of functional fibroblasts. Epidermal (cytokeratin 10, transglutaminase 1), dermo–epidermal junction (DEJ) (laminin 5, collagen-IV, collagen VII) and dermally-located (fibrillin 1, procollagen I) biomarkers were similar to those in NHS. Treatment of T-Skin™ with retinol decreased the expression of differentiation markers, cytokeratin 10 and transglutaminase 1 and increased the proliferation marker, Ki67, in epidermis basal-layer cells. Vitamin C increased the expression of DEJ components, collagen IV and VII and dermal procollagen 1. Conclusions: T-Skin™ exhibits structural and biomarker location characteristics similar to NHS. Responses of T-Skin™ to retinol and vitamin C treatment were consistent with those of their known anti-aging effects. T-Skin™ is a promising model to investigate responses of epidermal, DEJ and dermal regions to new skin anti-ageing compounds.

## 1. Introduction

Technological advances in simplified in vitro models representing in vivo organs or systems have enabled more complex target systems to be investigated experimentally and related responses to be predicted more accurately. The usefulness of any model is dependent on its characterization vis-à-vis the real system it aims to mimic, and the identification of differences, similarities and the applicability domain. Despite an apparent simplicity, skin is in fact a complex organization of cells and structures which are responsible for several essential functions for body homeostasis. Normal human skin (NHS) is composed of two main layers, the epidermis and the dermis, connected tightly by the dermo–epidermal junction (DEJ). The epidermis, primarily composed of keratinocytes, is a keratinized stratified squamous epithelium, characterized by continuously renewing cells to ensure maintenance of a functional barrier. The dermis, usually presented as a support tissue giving resistance and elasticity to skin, is mainly composed of an extracellular matrix (ECM) in which specialized cells, mainly fibroblasts, are embedded. The DEJ comprises a complex network of proteins and proteoglycans which connects the two layers and ensures important regulatory functions such as determining the polarity of the basal keratinocytes and contributing to wound-healing re-epidermization [1].

Reconstructed human epidermal (RHE) models have been developed to reproduce in vitro skin functions for research and evaluation purposes. The commercial availability of the SkinEthic™ RHE and EPISKIN™ RHE models in the early 1990s and their validations for use in skin irritation and corrosion assays [2,3] resulted in their wider use by the chemical, pharmaceutical and cosmetic industries. The implementation and harmonization of such methods in a regulatory context required a worldwide availability of reproducible RHE models. A key factor to achieve this was the capacity to scale up the production in a standardized manner. In parallel to the development of RHE models, efforts were made to add a living dermal compartment to produce models referred to as “reconstructed skin” or “full-thickness” models. Based on the pioneering work of Bell et al. [4], who developed a dermal equivalent derived from fibroblasts embedded in a collagen matrix, it was possible to seed keratinocytes directly on to the surface of the formed dermal lattice layer [5,6]. Although more complex to produce than RHE models, the presence of the living dermis extended the applicability domain to other areas of dermatological research. For example, full-thickness models allowed the investigation of ultraviolet-A-induced aging [7,8], and the role of papillary and reticular fibroblast populations [9], as well as glycation in aging to be deciphered [10,11]. Epidermal-dermal crosstalk is key for skin homeostasis; therefore, a model reflecting both epidermal and dermal functions is undoubtedly a driver in fostering innovative applications. Indeed, two other full-thickness models, PhenionFT and EpidermFT, have been used for different applications, including: environmental and age-dependent effects [12,13,14]; skin penetration [15,16]; ultraviolet (UV) irradiation effects [17,18,19]; skin metabolism [20]; genotoxicity [21,22]; wound healing [23,24,25]; disease mechanisms [26] and skin sensitization [27].Using the technology initially developed for research purposes [28], we have adapted the process for mass production of a new full thickness skin model, namely T-Skin™ (“T” refers to “total”, formerly RealSkin), according to ISO9001 standards to achieve reproducibility. The industrial-scale production of this reliable full-thickness model will allow for routine testing, as well as the development of research applications in academic and industrial scientific communities. To support the implementation of T-Skin™, we present a comprehensive characterization of the T-Skin™ epidermal and dermal compartments.

The second part of our studies was to evaluate whether T-Skin™ could be used for investigating anti-aging ingredients such as vitamin C or retinol. Protective effects of compounds against UV radiation-induced skin aging have also been studied in the PhenionFT model. They measured early effects according to changes in the gene expression profile [17]. Here, we evaluated beneficial effects of chemicals based on histochemistry and immunostaining of critical markers present in the skin models. Chronological aging is a complex biological phenomenon that affects all organs. In skin, this is evident as a decline in epidermal renewal, thinning of the epidermal layers, flattening of the DEJ, as well as a decrease in the number and activity of fibroblasts. Exogenous parameters can impact these changes e.g., solar UV induces photo-aging signs such as solar elastosis. Among the strategies developed to limit the effects of aging, topical applications of antioxidants have been shown to be beneficial by quenching free radicals [29]. Vitamin C is used extensively in cosmetics for its potent antioxidant properties and beneficial effects on skin including induction of collagen neo-synthesis, inhibition of melanogenesis and improvement of a variety of inflammatory skin disorders [30,31,32]. Alterations of the DEJ and dermis are reported to occur during chronological ageing and vitamin C is known to improve the quality of the skin ECM [33]. Therefore, full thickness models have an added value in assessing the efficacy of anti-aging ingredients and finished products, which can act not only on the upper layers of the skin (epidermis) but target also the dermis.

## 2. Results

### 2.1. Characterization of T-Skin™ Model

#### 2.1.1. Time Course of T-Skin™ Model Reconstruction

**Figure 1:**
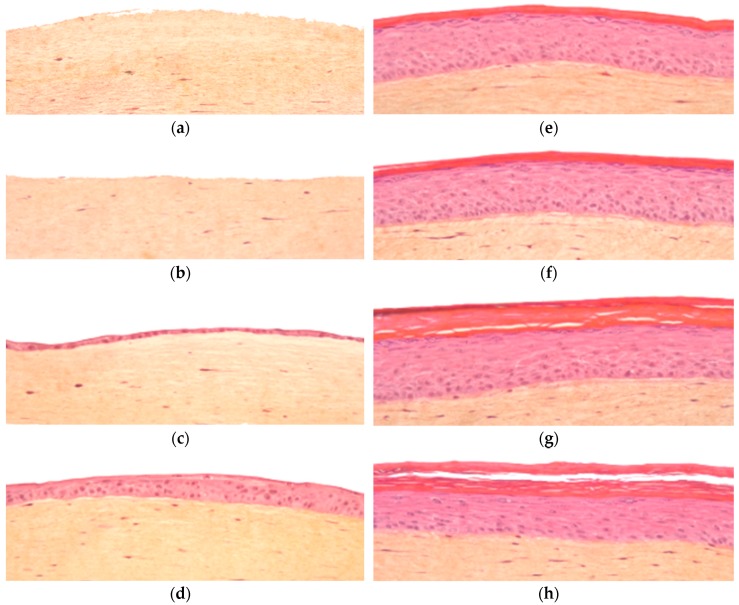
Kinetic of T-Skin™ model histology studied by hematoxylin-eosin-safran staining over 25 days: (**a**) day 1; (**b**) day 5 (**c**) day 8; (**d**) day 13; (**e**) day 18; (**f**) day 20; (**g**) day 22 and (**h**) day 25.

The time course of the formation the dermal equivalent and the subsequent addition, growth and differentiation of the epidermal layer of T-Skin™ is shown in Figure 1. During the first 5 days, the collagen I reticulates to form a lattice mimicking dermal extracellular matrix with living homogenously distributed fibroblasts. After 5 days, keratinocytes seeded on the top of the dermal lattice form a thin layer, which thickens and differentiates gradually and evenly over the following 13 days to a maximum thickness of 8–12 living layers covered by a stratum corneum on Day 18 when the tissues are released and shipped to the user laboratory. The structures of the dermis and epidermis are maintained from day 18 until at least day 25, allowing for endpoint assays to be conducted during this time.

#### 2.1.2. Epidermal Characterization

**Figure 2:**
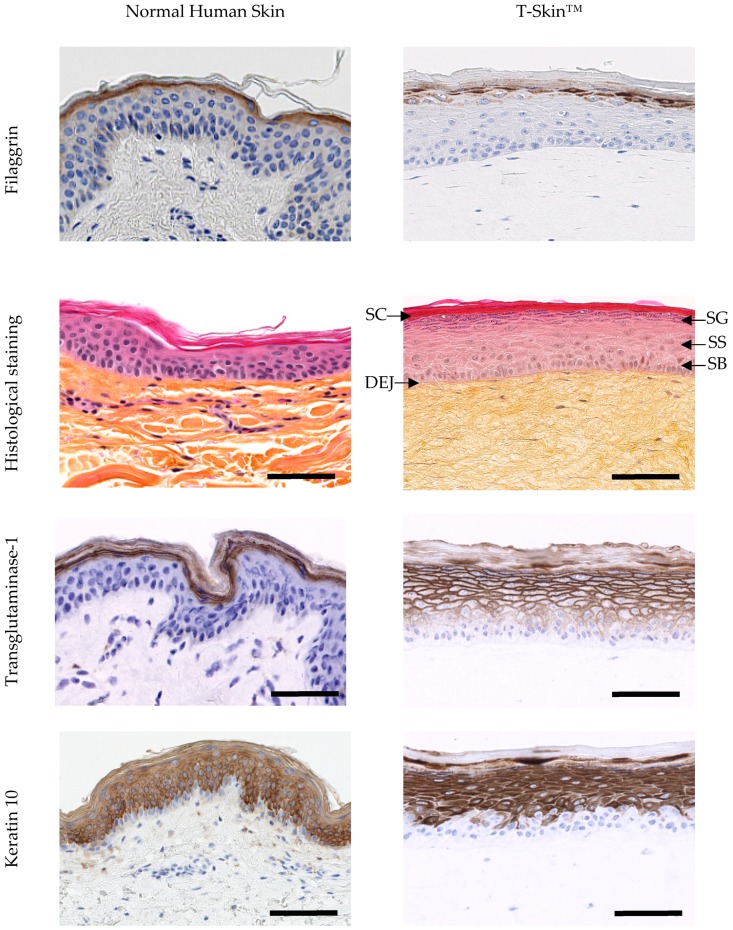

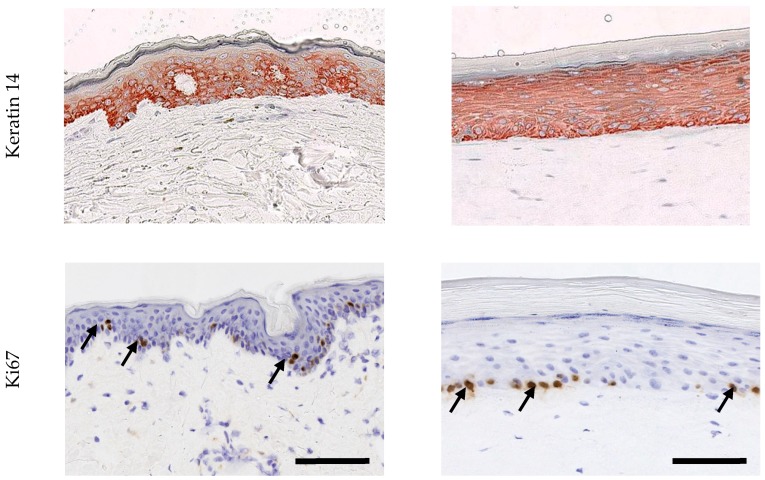
Characterization of the epidermis from normal human skin (NHS) and T-Skin™ by histology and immunohistochemistry analysis on paraffin sections. Localization on histological stained images of the stratum corneum (SC), stratum granulosum (SG), stratum spinosum (SS), stratum basal (SB) layers of the epidermis and the dermo–epidermal junction (DEJ). For histological staining, the scale bar represents 100 μm. All other bars are 100 μm. Arrows indicate positive cells for Ki67 expression

Histological images show a good similarity in the organization and differentiation of the epidermal compartment in T-Skin™ and NHS (Figure 2). The main epidermal layers known to occur in vivo, namely, the stratum basal, spinosum, granulosum and corneum, are visible in T-Skin™ (denoted in Figure 2). The expression profiles of 6 epidermal markers reflecting the differentiation process are shown in Figure 2. The proliferative activity of the keratinocytes, restricted to the basal layer, is detected by the immuno-labelling of Ki67, a protein accumulated within the nucleus during mitosis. The spatio-temporal differentiation process, which occurs in the epidermis, is reflected by the localization of different biomarkers. Keratin 14 is mainly expressed in the basal layers but can also be observed in supra-basal layers, depending of the anti-body used [34]. Keratin 10, a constitutive feature of the intermediate filaments and not synthetized in the basal layer, appears in the keratinocytes of the spinous layers and extends to all the suprabasal layers. These two markers were similarly expressed in NHS and T-Skin^TM^. The membrane enzyme, transglutaminase 1, an intermediate marker of epidermal differentiation that participates in the formation of the cornified envelop of corneocytes, is mainly located in the granular layer in NHS, while in T-Skin™, it is also expressed earlier in the suprabasal layers. Filaggrin, a marker of late differentiation, is present in the keratohyalin granules of the granular layer in the form of profilaggrin and as filaggrin in the corneal layer. This is localized in the same area in NHS and T-Skin^TM^.

#### 2.1.3. Dermo–Epidermal Characterization

**Figure 3:**
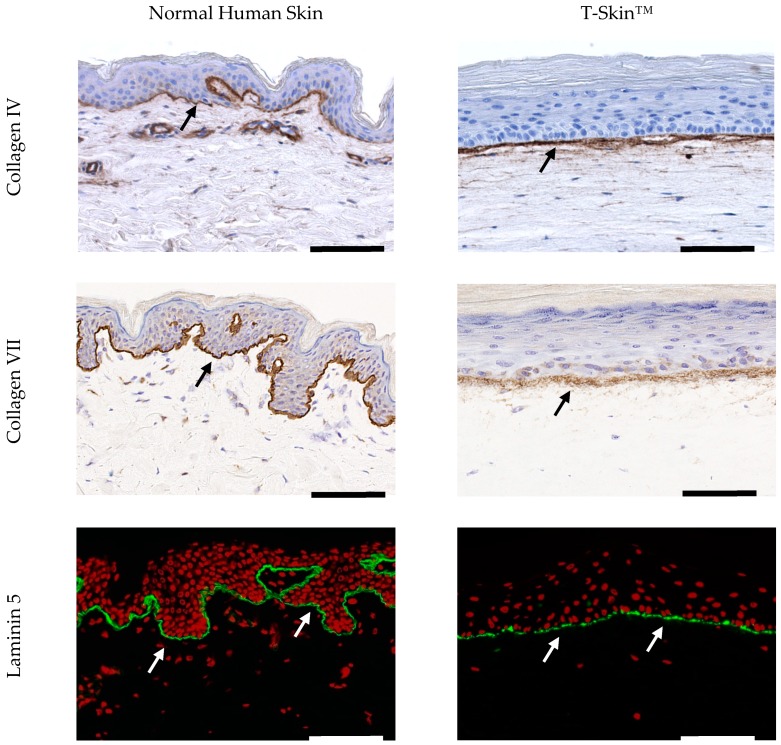
Characterization of the DEJ in NHS and T-Skin™. Collagen IV and VII immunohistochemistry analysis was performed on paraffin sections and laminin-5 immunofluorescence analysis was performed using cryostat sections. Arrows indicate the localization of specific labelling; scale bars indicate 100 μm.

Figure 3 shows the major components of the DEJ expressed in NHS and T-Skin™. Collagen IV and laminin-5 belong to the complex network of proteins and proteoglycans which connect the two layers and ensures important regulatory functions. The early phase of DEJ formation involves the assembly of laminins through binding to cell membrane receptors, as well as a scaffold formation of type IV collagen. Collagen VII is an anchoring fibril collagen binding with type I and type III collagen. Collagen IV and VII, as well as laminin-5, were clearly detected at the DEJ of T-Skin™ and their locations in the tissue were similar to that of NHS.

#### 2.1.4. Dermal Characterization

**Figure 4:**
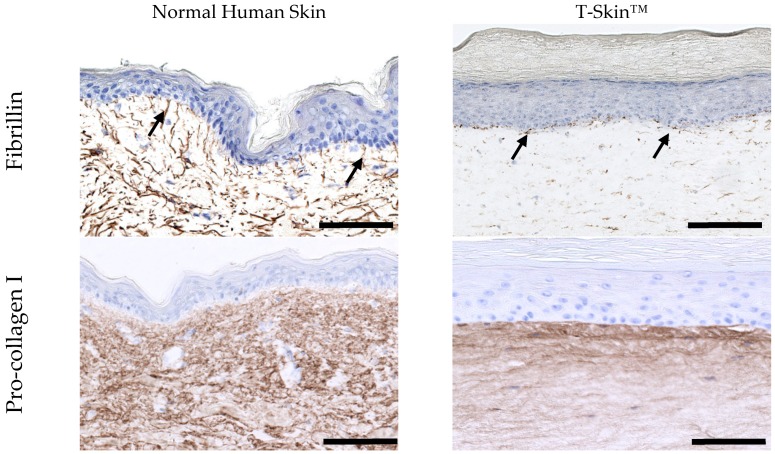
Characterization of the dermis of NHS and T-Skin™. Immunohistochemistry of fibrillin 1 and pro-collagen I was performed using on paraffin sections. Arrows indicate the localization of the specific labelling. Bars = 100 μm.

The dermal layer of T-SkinT™ is populated with primary fibroblasts with a density close to that observed in NHS, as shown in histological images in Figure 1 and Figure 2. The expression of fibrillin 1 and pro-collagen I in the dermal layer of NHS and T-Skin™ is shown in Figure 4. Fibrillin 1 is a structural glycoprotein to which molecules of tropo elastin are attached to form dermal elastin fibers. Elastin is not clearly detected in T-Skin™ but newly synthetized fibrillin 1 is detected in the whole dermis. The expression is higher in the upper layers, close to the DEJ. In parallel, pro-collagen I, the precursor of collagen I, is readily detected across all layers of the dermis of NHS and T-Skin™. As with fibrillin 1, more intense labelling is observed in the upper dermis near the DEJ.

### 2.2. Application of T-Skin™ to Anti-Aging Investigations

#### 2.2.1. Effect of Retinol on Epidermal Markers

**Figure 5:**
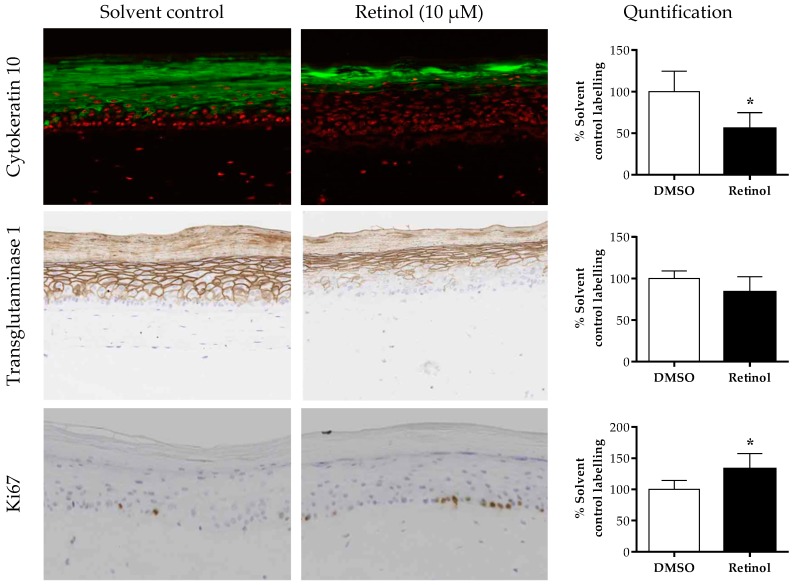
Epidermal effect of retinol on different biomarkers. T-Skin™ models were treated with 10 µm retinol or 0.1% DMSO for 5 days. The intensity of the fluorescence or DAB staining of immunolabeled section of the T-Skin™ model was assessed by a semi-quantitative scoring method for the differentiation epidermal markers, cytokeratin 10 and transglutaminase 1. For the proliferative marker, Ki67, the number of red nuclei in the basal membrane were counted. The results are expressed as a percentage of control values, mean ± standard error of the mean (SEM); *n* = 3 T-Skin™ batches. A *p* value of < 0.05 was considered to be statistically significant, denoted by an asterisk.

The anti-aging effects of 5 days of treatment with 10 μM retinol were tested in T-Skin™. After retinol treatment (a non-cytotoxic concentration, Supplementary Table S1A), there was a decrease in the expression of the differentiation markers, cytokeratin 10 and transglutaminase 1, compared to solvent control-treated tissues (Figure 5). This is expressed semi-quantitatively using image analysis, indicating a statistically significant decrease of 45% (*p* = 0.006) in cytokeratin 10 expression and a small but non-significant decrease of 17% (*p* = 0.08) in transglutaminase 1 expression. Moreover, after treatment with retinol, cytokeratin 10 expression is delayed and was detected in the epidermal granulosum layer instead of the granulosum and spinosum layers observed in control-treated tissues. In parallel, there was a statistically significant increase of Ki67 (132% of control treated tissue, *p* = 0.02) in the basal layer of the epidermis after retinol treatment.

#### 2.2.2. Effect of Vitamin C on Dermo–Epidermal and Dermal Markers

**Figure 6:**
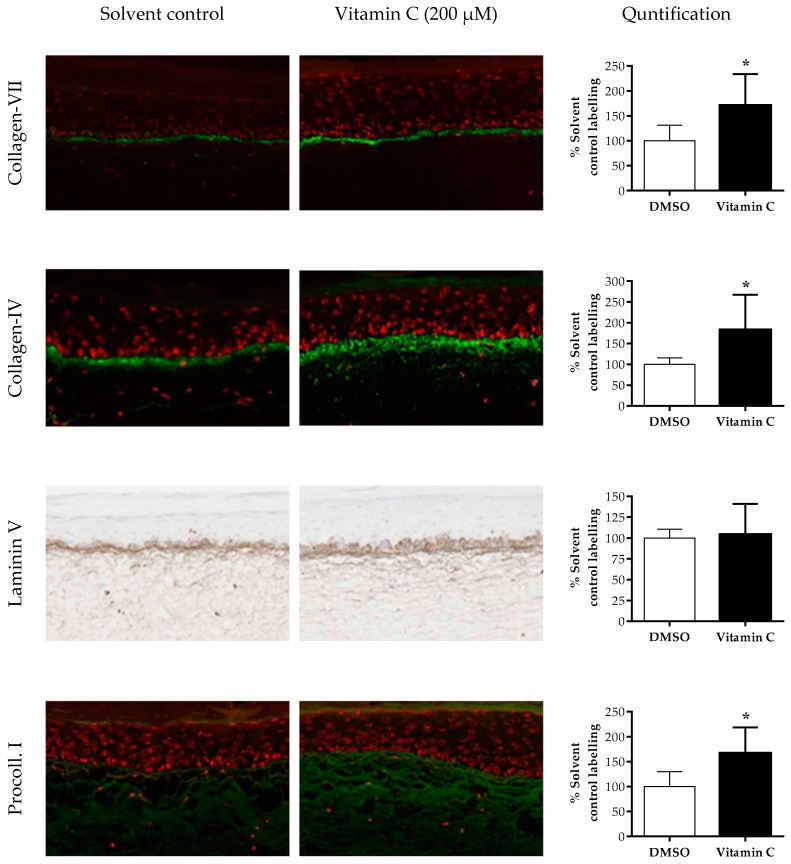
Effects of vitamin C on different biomarkers in the dermis and DEJ of T-Skin™. Models were treated with 200 µm vitamin C or 100% water for 5 days. The DEJ markers collagen IV, VII and laminin 5 were analyzed. The pro-collagen 1 marker was analyzed in the dermal compartment only. The intensity of the fluorescence or DAB staining of immunolabeled sections of T-Skin™ was assessed using a semi-quantitative scoring method. The quantified results are expressed as a percentage of control treated values, mean ± SEM; *n* = 3 T-Skin™ batches. A *p* value of < 0.05 was considered to be statistically significant, denoted by an asterisk.

The anti-aging effects of vitamin C were determined using T-Skin™. The expression of four matrix components was measured after treatment with 200 μM vitamin C (non-cytotoxic concentration, Supplementary Table S1B) for 5 days (Figure 6). Collagen VII and IV expressions were statistically significantly increased by vitamin C by 170% (*p* = 0.03) and 183% (*p* = 0.04) of control, respectively. The level of expression of laminin-5 was not statistically significantly altered (*p* = 0.79) but the spread of labelling of this marker was more diffuse on each side of the DEJ. The expression of pro-collagen 1 was increased by vitamin C treatment, with an increase of 167% of control-treated tissues.

## 3. Discussion

Our results show that T-Skin™ exhibits a very similar structure to NHS, composed of a living epidermis of primary keratinocytes and a living dermis with primary fibroblasts seeded in a collagen type I gel. The main notable structural difference was the flat shape of the DEJ in the reconstructed model, whereas in NHS rete ridges are present. These are epidermal extensions going downward into the dermis, to fit the shape of dermal extensions (dermal papilla). Some in vitro models reconstructed on de-epidermized dermis preserve this undulatory structure of the DEJ [35]; however, these have some important limitations: firstly, only the epidermis contains living cells and, secondly, they cannot be produced at an industrial scale due to limited availability of human dermis.

The organization and differentiation of the epidermis in T-Skin™ and NHS were similar, both exhibiting the typical basal, spinosum, granulosum and stratum corneum layers. The deepest layers comprising the basal or germinal layers are where keratinocytes multiply and renew the epidermal layers [36]. This proliferative activity was detected by immuno-labeling of the nuclear-located Ki67 protein in the basal layer of NHS and T-Skin™. Newly formed keratinocytes migrate upwards and undergo a programmed differentiation process that ultimately leads to their death when transformed into corneocytes [37]. Each stage of epidermal differentiation is characterized by the expression of specific proteins. In the spinous layer, the cells flatten and differentiation-specific proteins, such as keratin 10, are expressed. Keratin 10 was present in NHS and T-Skin™ in all suprabasal layers, albeit with a slight delay in the appearance in T-Skin™. The next layer, the granular layer, is characterized by flattened cells containing keratohyalin granules, which were clearly visible in both T-Skin™ and NHS. The uppermost stratum corneum layer is composed of corneocytes organized in a “brick and mortar”-like structure which ensures the barrier function of the epidermis [38]. Transglutaminase 1, an essential enzyme for a correct cornified envelop formation [39], is preferentially associated to the membrane of the granular cells [40] and was present in this layer in T-Skin™ and NHS, although this marker is also detected in deeper layers of the T-Skin™ model. Filaggrin is a marker of late differentiation and is present in the keratohyalin granules of the granular layer in the form of profilaggrin (formed from multiple copies of filaggrin). In the corneal layer, the granules disintegrate and profilaggrin is converted into filaggrin. Filaggrin is then able to aggregate the intermediate keratin filaments of the tonofilaments into an organized network essential to the mechanical strength of the corneal layer. The observed localization of Filaggrin in NHS and T-Skin^TM^ is in accordance with these late mechanisms of differentiation.

The dermis of T-Skin™ is populated with primary fibroblasts to mimic NHS. In vivo, fibroblasts synthesize and renew the ECM, which is composed of a mixture of water and proteoglycans in a dense network of fibrillary proteins. Type I collagen is the main component of this fibrillary network [41] and is also the component of the lattice of the T-Skin™ dermis. In vitro, the abundance of existing collagen I limits detection of de novo synthesis of this protein. For this reason, we selected pro-collagen I, a precursor of collagen I, to study fibroblast functional activity in the ECM. The intense labelling of pro-collagen I in the T-Skin™ dermis confirmed the capacity of fibroblasts to synthetize new ECM components. Elastin is another fibrillar component of the ECM which confers its elastic properties to the dermis [42]. Elastin was not detected in T-Skin™ but new synthetized fibrillin was detected in the entire dermis layer, with a higher quantity in the superficial dermis, close to the DEJ. In vivo, this region, called papillary dermis, is characterized by the presence of thin elastic oxytalan fibers oriented perpendicularly to the DEJ [43]. Basal keratinocytes are thought to participate in the microfibril synthesis in this region [44], which could explain the intense fibrillin staining of the DEJ in T-Skin™.

DEJ formation requires the participation of both keratinocytes and fibroblasts to regulate the process [45]. The early phase of DEJ formation involves the assembly of laminins through binding to cell membrane receptors, as well as a scaffold formation of type IV collagen [46]. These two markers are detected at the junction of the T-Skin™ epidermis and dermis, indicating the capacity of this model to synthesize the natural constituents of the DEJ.

The second part of the study aimed at assessing the responses of the T-Skin™ model after treatments with anti-aging compounds, retinol and vitamin C. Both compounds are used clinically for the treatment of photo-aging [47], making them suitable test compounds for this investigation. The doses of retinol and vitamin C were non-cytotoxic according to MTT measurements and the treated T-Skin™ continued to exhibit a well-developed and organized epidermis with no observable alteration in morphology. In vivo, retinol modulates epidermal differentiation and proliferation [48], two important mechanisms disturbed in the ageing of skin. These studies showed that the same processes were modulated in the same way in T-Skin™. Indeed, retinol stimulated cell proliferation, as reflected by the increase in Ki67 labelling in the epidermal basal layer of T-Skin™. At the same time, keratinocytes differentiation was delayed, reflected by the decrease in transglutaminase and keratin 10 labelling.

In the dermis, skin aging is associated with a decrease of collagens resulting from both degradation of the existing ECM and failure of fibroblasts to synthetize new pro-fibrillar proteins, such as pro-collagen I [49]. In vivo, topical treatment with vitamin C counteracts these alterations by stimulating collagen synthesis within the ECM [50]. This beneficial effect of vitamin C was also demonstrated using T-Skin™, in which there was an increase in the labelling of pro-collagen I. In-vivo, aging also affects the DEJ, with a decrease of its main component, collagen IV [51]. As expected, vitamin C also stimulated the expression of several constitutive proteins of the basal membrane in T-Skin™, such as collagen IV and collagen VII, but not laminin 5 [52,53]. Another sign of skin aging and photo-aging is the decrease in the papillary dermis of the presence of vertically oriented, thin, elastic (oxytalan) fibers [54]. Likewise, vitamin C also caused an increased fibrillin expression in the upper region of the equivalent dermis of T-Skin™, suggesting a beneficial stimulating effect.

In conclusion, this study shows that the T-Skin™ model exhibits a number of NHS characteristics, including a well-differentiated and organized epidermis and a functional dermis. Beneficial effects of retinol and vitamin C on the different layers of T-Skin™ are in agreement with their known skin anti-aging properties in vivo, underlining the predictive capacity of this model. Investigations on aging markers using T-Skin™ and subsequent human clinical evaluation have already been used successfully for the development of a new cosmetic anti-aging active ingredient [55]. These results suggest that T-Skin™ could be an interesting and predictive model to investigate the anti-aging effects of compounds on the epidermal, DEJ and dermal layers. In conclusion, our results support the use of T-Skin™, produced in a mass scale, as a powerful tool in a screening platform to develop new cosmetics and dermatologically active ingredients.

## 4. Materials and Methods

### 4.1. T-Skin™ Preparation

Keratinocytes and fibroblasts were isolated from skin obtained from breast plastic surgery after informed patient and her written consent. The dermal equivalent was produced by culturing normal human fibroblasts with a lattice of native collagen type I containing for 5 days at 37 °C under 5% CO_2_. After 5 days, keratinocytes were seeded on the top of the dermal lattice and cultured for a further 13 days in the appropriate culture medium. A well stratified and fully differentiated epidermis is obtained after these 18 days of cultivation. T-Skin™, is manufactured according to ISO9001 quality system and each batch is controlled according to EPISKIN SA (Lyon, France) standard quality control criteria.

### 4.2. Histological and Immunohistological Characterization

Normal human skin (NHS) and T-Skin™ models were fixed in 10% neutral-buffered formalin, dehydrated in graded alcohols then xylene, and embedded in paraffin blocks to obtain formalin-fixed paraffin-embedded (FFPE) blocks (formalin-fixed paraffin-embedded block). Four-micron thick sections were cut on a rotary microtome from FFPE blocks, and these tissue sections were rehydrated in xylene then alcohols.

The hematoxylin-eosin-safran staining was performed on paraffin sections using the Sakura Tissue-Tek^®^ Prisma^®^ automated slide stainer, according to the manufacturer’s protocol.

Immunohistochemical staining was performed using the Omnis automated staining system (Agilent Technologies, Santa Clara, CA, USA) according to the manufacturer’s instructions. Antigen retrieval was performed using the EnVision FLEX Target Retrieval Solution. Tissue sections were incubated with following primary antibodies: transglutaminase 1 (clone BT-621, Biomedical Technologies, Stoughton, MA, USA); keratin 14 (clone LL002; ABCAM); keratin 10 (clone DE-K10), Ki67 (clone MIB-5) and collagen IV (clone CIV 22) (from Agilent); fibrillin (clone 26, Millipore); laminin 5 (clone P3H9-2) and pro-collagen I (clone 2Q576) (from Abcam, Cambridge, MA, USA). After the chromogenic visualization step using the EnVision FLEX diaminobenzidine (DAB), slides were counterstained with hematoxylin and covered with a coverslip. Appropriate positive and negative controls were stained concurrently to validate the staining procedure.

T-Skin™ and NHS were labelled with immunofluorescence as positive controls. Non-fixed tissues, embedded in Tissue-Tek O.C.T. (Sakura, Villeneuve d’Ascq, France), were snap-frozen in liquid nitrogen. Cryostat sections of 7 µm thickness were cut and incubated with the following primary antibodies: filaggrin (clone AKH1) and collagen VII (clone 4D2Santa) (Cruz Biotechnology, Santa Cruz, CA, USA), transglutaminase 1 (clone B.C1; Biomedical Technologies Inc., Stoughton, MA, USA); keratin 10 (clone RKSE60; Monosan, Uden, Netherlands); Ki67 (clone MIB1; Agilent, Billerica, MA, USA); collagen IV (clone CIV 22; Agilent, Santa Clara, CA, USA); laminin-5 (clone D4B5), procollagen I (clone M-58) and fibrillin (clone 11C1.3) (Millipore, Billerica, MA, USA). Staining was visualized using the appropriate secondary antibody conjugated to Alexa488^®^ (Life Technologies, Grand Island, NY, USA). Nuclei were stained with propidium iodide (Sigma-Aldrich, St Louis, MO, USA).

### 4.3. Cytotoxicity Measurement

Cytotoxicity was determined using the MTT conversion test on separated dermal and epidermal compartments. Intact T-Skin™ models were incubated with 2 mL of MTT (1 mg/mL) for 3 h at 37 °C, 5% CO_2_, 95% air. At the end of incubation, the dermis and epidermis were gently separated and formazan extractions of these compartments were performed separately with 900 μL isopropanol. Absorbance was measured at 570 nm. Tissues viabilities were expressed as a percentage of control (solvent-treated) samples.

### 4.4. Effect on Skin Aging Markers

T-Skin™ models were received in the user laboratory on Day 18 of culture and maintained for a further 24 h in fresh maintenance medium at 37 °C under 5% CO_2_. Retinol (68-26-8; Sigma-Aldrich, St Louis, MO, USA) at 10 µM, DMSO 0.1% (retinol control), vitamin C (200 µM (113170-55-1; Sigma-Aldrich) or water (vitamin C control) were added to the culture medium and incubated for 5 days. The medium containing compounds was renewed every 48 h. At the end of the treatment, 4 µm paraffin or cryostat sections were performed for immunofluorescence or immunohistochemical applications. The biological effects of vitamin C were evaluated on the DEJ and dermal biomarkers (laminin 5, procollagen I, collagen IV and VII). The effects of retinol were evaluated using the epidermal biomarkers, Ki67, transglutaminase 1 and cytokeratin 10. Effects of the compounds compared to their respective control were assessed using a semi-quantitative scoring method of the immuno-labelled sections where 0 = no staining and 4 = maximal staining. The scale incorporated the intensity and extent of specific markings. Results represent the mean of 3 inserts, replicated on 3 tissues batches, 4 sections were analyzed per insert and 3 fields per section.

### 4.5. Statistical Analyses

The effects of retinol and vitamin C on the different markers were compared to control-treated models using a two-tailed T-test using GraphPad Prism Version 6.07. A *p* value of >0.05 was considered to be statistically significant.

## Supplementary Materials

Supplementary materials can be found at https://www.mdpi.com/1422-0067/20/9/2240/s1.

## Abbreviations

NHS	normal human skin
DEJ	dermo-epidermal junction
ECM	extracellular matrix
RHE	reconstructed human epidermal
FFPE	formalin-fixed paraffin-embedded block
SC	stratum corneum
SG	stratum granulosum
SS	stratum spinosum
SB	stratum basal
