# Supplementary material for: Characterization of a New Reconstructed Full Thickness Skin Model, T-Skin™, and its Application for Investigations of Anti-Aging Compounds
Source: Int J Mol Sci. 2019 May 7;20(9):2240. doi: 10.3390/ijms20092240 (PMC6540298; doi:10.3390/ijms20092240)
Supplement: Supplementary file 1 [file ijms-20-02240-s001.pdf]

Supplementary Table 1: Viability of T-Skin™ models with and without treatment of (A) 200 μM vitamin C and (B) 10 μM retinol. Values are expressed as mean ± SD of the % untreated model metabolism of MTT in intact models and separated epidermis and dermis layers.

(A) Vitamin A

	Whole skin	Epidermis	Dermis
Untreated	100.0 ± 0.0	100.0 ± 0.0	100.0 ± 0.0
Vitamin C (200 μM)	99.0 ± 18.0	99.6 ± 17.2	117.8 ± 14.9

(B) Retinol

	Skin	Epidermis	Dermis
DMSO 0,1%	100.0 ± 3.9	100.0 ± 4.7	100.0 ± 1.1
Retinol 10 μM	107.9 ± 7.1	111.4 ± 7.2	95.1 ± 9.5
